# Unresectable Foramen Magnum Meningioma Treated With CyberKnife Robotic Stereotactic Radiosurgery

**DOI:** 10.7759/cureus.8409

**Published:** 2020-06-02

**Authors:** Janna Malone, Eduardo Gaviolli, Janice Doody, John Sinclair, Shawn Malone

**Affiliations:** 1 Radiation Oncology, The Ottawa Hospital, Ottawa, CAN; 2 Medicine, McMaster University, Hamilton, CAN; 3 Radiation Oncology, Dr. H Bliss Murphy Cancer Centre, St. John's, CAN; 4 Neurosurgery, The Ottawa Hospital, Ottawa, CAN

**Keywords:** meningioma, foramen magnum, cyberknife, radiosurgery

## Abstract

Meningiomas are tumors that originate from the meningeal or dural cover of the brain and are the most common primary benign brain tumors. Currently, the accepted management of these tumors is attempted total surgical excision when technically possible and associated with an acceptable risk. However, radiation therapy has been shown to provide excellent local control when used either as an adjunct to surgery or as a primary treatment. We present a case report of a 46-year-old female patient with an unresectable left foramen magnum meningioma resulting in headaches, neck pain, and swallowing difficulty. The patient underwent CyberKnife (Accuray Incorporated, Sunnyvale, CA) radiosurgery to a dose of 3,000 cGy in five fractions in March 2011. The patient tolerated treatment without complications and has remained clinically well with no ongoing cranial nerve deficits as of the last examination in late 2019. This demonstrates the excellent local control obtained when using radiosurgery as both a surgical adjunct and a primary treatment for meningiomas.

## Introduction

Meningiomas are tumors that originate from the meningeal or dural cover of the brain. Meningiomas are the most common benign primary brain tumor, accounting for approximately 25% of all intracranial neoplasms, which increased to 40% if autopsy data are included [1]. The vast majority of these tumors are benign, WHO grade 1 tumors. They do, however, have the potential to transform into atypical (WHO 2) or anaplastic (WHO 3) tumors, as is seen in 5%-15% and 1%-2% of all meningioma cases, respectively. Meningiomas may occur spontaneously or as a result of previous ionizing radiation, neurofibromatosis type 2, and other genetic syndromes [2]. Incidence rates of meningiomas increase with age, with a dramatic increase in adults aged 65 years and older, and are much more common in women than in men. Meningiomas are found incidentally at rates of up to 50% of confirmed diagnosis, but symptomatic presentation is dependent on size and location of the tumor. While some meningiomas may not grow, annual growth rates have been reported between 0.6% and 21% [3].

## Case presentation

A 46-year-old female patient presented with a one-year history of progressive left occipital headaches and neck pain, with a six-month history of difficulty swallowing and a thickening sensation in her tongue. She also reported some difficulties with speech and a heightened sensitivity in the left V1/V2 trigeminal distributions, with referred otalgia. MRI completed in July 2010 revealed a mass in the left foramen magnum, isointense on T1 and T2 and enhancing with gadolinium, compressing the left side of the medulla and upper cervical cord, and extending through the left occipital bone and hypoglossal canal to the left jugular foramen region and left carotid sheath. The patient was initially seen at a more remote neurosurgery center, following which the patient was referred for a multidisciplinary assessment at a tertiary care center with expertise in skull base surgery. The tumor was deemed inoperable due to high morbidity associated with the location and extent of the tumor. The patient was referred to our center for consideration of CyberKnife (Accuray Incorporated, Sunnyvale, CA).

On examination in January 2011, she was found to have increased sensitivity in the V1/V2 distributions and impaired mobility of the tongue resulting in dysarthria. A pre-procedure swallowing study revealed no oral or pharyngeal swallowing difficulties; however, the patient subjectively had some difficulty swallowing both solids and liquids.

**Figure 1:**
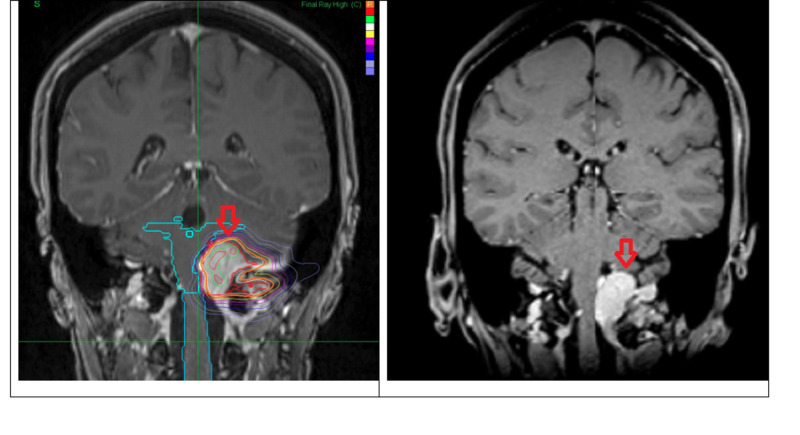
Left: pre-treatment coronal view of T1 contrast-enhanced MRI. Right: eight years post-treatment showing a 30% decrease in tumor volume and involution of mass effect on the brainstem.

In March 2011, the patient underwent CyberKnife radiosurgery to a dose of 3,000 cGy in five fractions. Treatment was delivered using CyberKnife robotic stereotactic radiosurgery (SRS) with real-time tumor tracking (Figure 1). Radiation therapy was delivered by a 6 MV compact linear accelerator mounted on top of a robotic arm.

The patient tolerated treatment without complications and no pre-medication was required. Nine months post-radiosurgery, the patient had improvement in pain and no longer required opioids around the clock. Examination revealed relatively normal cranial nerve examination with good swallowing and gag reflex. Facial sensation was within normal limits.

The patient remained clinically well and as of the most recent examination in 2019, she continues to remain well with no cranial nerve deficits, a slightly deviated tongue, no swallowing issues, and normal gait (Figure 1).

## Discussion

Meningiomas may present with a wide array of symptoms dependent upon their size, location, and associated presence of vasogenic edema. Foramen magnum meningiomas represent a common histological tumor in a rare and eloquent location [4]. Surgery and more recently radiation therapy have been cornerstone treatments for meningiomas of all grades. Currently, the accepted management of these tumors is attempted total surgical excision when technically possible and when associated with an acceptable risk. In most patients, surgical resection is recommended to relieve neurological symptoms and gross total resection is often curative [5]. However, radiation therapy has been shown to provide excellent local control when used either as an adjunct to surgery or as a primary treatment with many radiosurgery series demonstrating local control rates of 92%-100% at five years and 88%-95% at 10 years [6]. This patient was treated with CyberKnife radiosurgery to a prescribed dose of 3,000 cGy in five fractions for an inoperable meningioma.

Pre-treatment volumetric data from 2011 showed a 14.2 cm^3^ enhancing meningioma. The most recent surveillance MRI in April 2019 showed a residual enhancing volume of 10 cm^3^, which is equivalent to 30% volumetric involution.

Considering the complex anatomy of the foramen magnum region and the encasement of anatomical structures by the tumor, surgical resection would result in extensive morbidity and result in subtotal resection. Radiosurgery is a well-established alternative to treat unresectable lesions. Given the mass effect of the tumor over the brainstem, it was elected to proceed with fractionated radiosurgery in order to respect the dose constraints for the brainstem.

The patient tolerated the treatments without issue and she started to improve clinically and radiographically within nine months post-treatment. She is currently eight years post-treatment continuing to present gradual and progressive clinical improvement and radiological regression of the residual enhancing lesion.

## Conclusions

The dramatic presentation and learning points pertaining to this case include the compression of the medulla and upper cervical cord, with tumor extending through the left occipital bone and hypoglossal canal to the left jugular foramen region and left carotid sheath. Tumor regression with symptom improvement and long-term local control were obtained with fractioned CyberKnife radiosurgery.
